# Supplementary material for: Differential Modulation of Human M1 and M2 Macrophage Activity by ICOS-Mediated ICOSL Triggering
Source: Int J Mol Sci. 2023 Feb 2;24(3):2953. doi: 10.3390/ijms24032953 (PMC9917690; doi:10.3390/ijms24032953)
Supplement: Supplementary file 1 [file ijms-24-02953-s001.zip › ijms-2119980-supplementary.pdf]

International Journal of *Molecular Sciences*

MDPI

Supporting Information

Differential Modulation of Human M1 and M2 Macrophage Activity by ICOS-Mediated ICOSL Triggering

Casimiro Luca Gigliotti [1,2,†], Chiara Dianzani [3,†], Ian Stoppa [1], Chiara Monge [3], Salvatore Sutti [1], Daniele Sblattero [4], Chiara Puricelli [1,5], Roberta Rolla [1,5], Umberto Dianzani [1,5,*,‡] and Elena Boggio [1,2,‡]

[1] Department of Health Sciences, Università del Piemonte Orientale, 28100 Novara, Italy
[2] NOVAICOS s.r.l.s, Via Amico Canobio 4/6, 28100 Novara, Italy
[3] Department of Scienza e Tecnologia del Farmaco, University of Turin, 10125 Turin, Italy
[4] Department of Life Sciences, University of Trieste, 34127 Trieste, Italy
[5] Clinical Biochemistry Laboratory, Maggiore della Carità University Hospital, 28100 Novara, Italy
* Correspondence: umberto.dianzani@med.uniupo.it; Tel.: +39-032-1373-3487
† These authors contributed equally to this work.
‡ These authors contributed equally to this work.

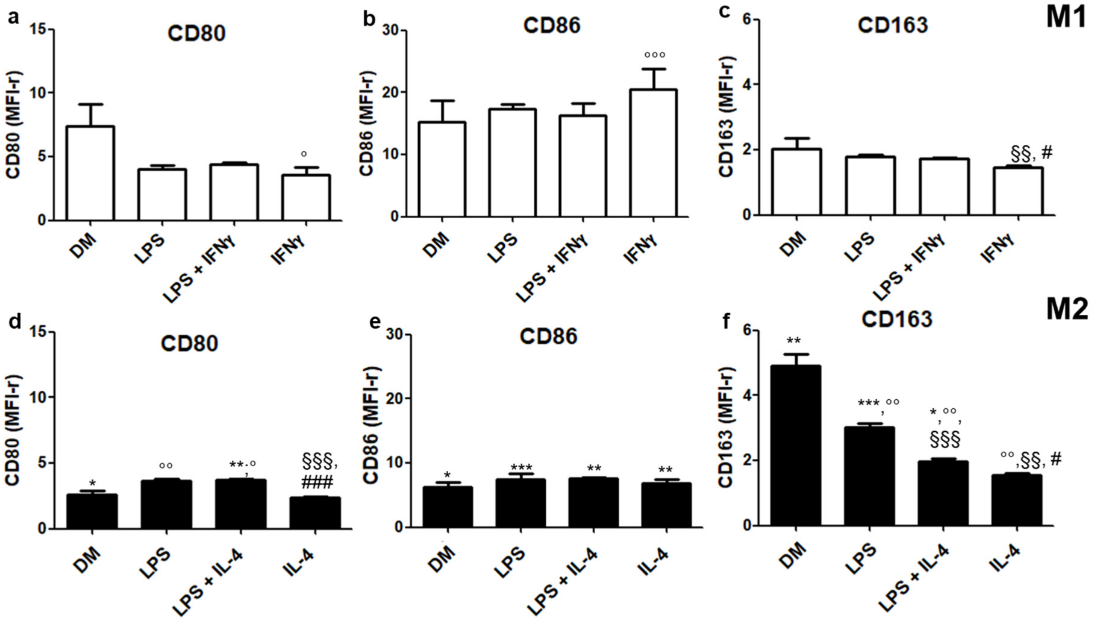

Figure S1. CD80, CD86, and CD163 expression on M1 and M2 cells. Monocytes were cultured in differentiation medium (DM), (GM-CSF for M1 polarization or M-CSF for M2 polarization). CD80 (**a**–**d**), CD86 (**b**–**e**), and CD163 (**c**–**f**) expression was evaluated after 6 days (**d**) (T6) of culture in the presence of GM-CSF or M-CSF alone or after an additional 48 hours (h) (T6 + 2) in the presence of LPS, IFNγ, or LPS+IFNγ (M1) or LPS, IL-4, or LPS+IL-4 (M2) by flow cytometry. Data represent the mean and SEM expressed as mean fluorescence intensity ratio (MFI-R) of results from five independent experiments. * $p < 0.05$, ** $p < 0.01$, *** $p < 0.0001$ versus the corresponding M1; §§ $p < 0.01$, §§§ $p < 0.0001$ vs LPS or ° $p < 0.05$, °° $p < 0.01$, °°° $p < 0.001$ vs. DM; # $p < 0.05$, ### $p < 0.0001$ vs. LPS+ IFNγ or LPS+IL-4.

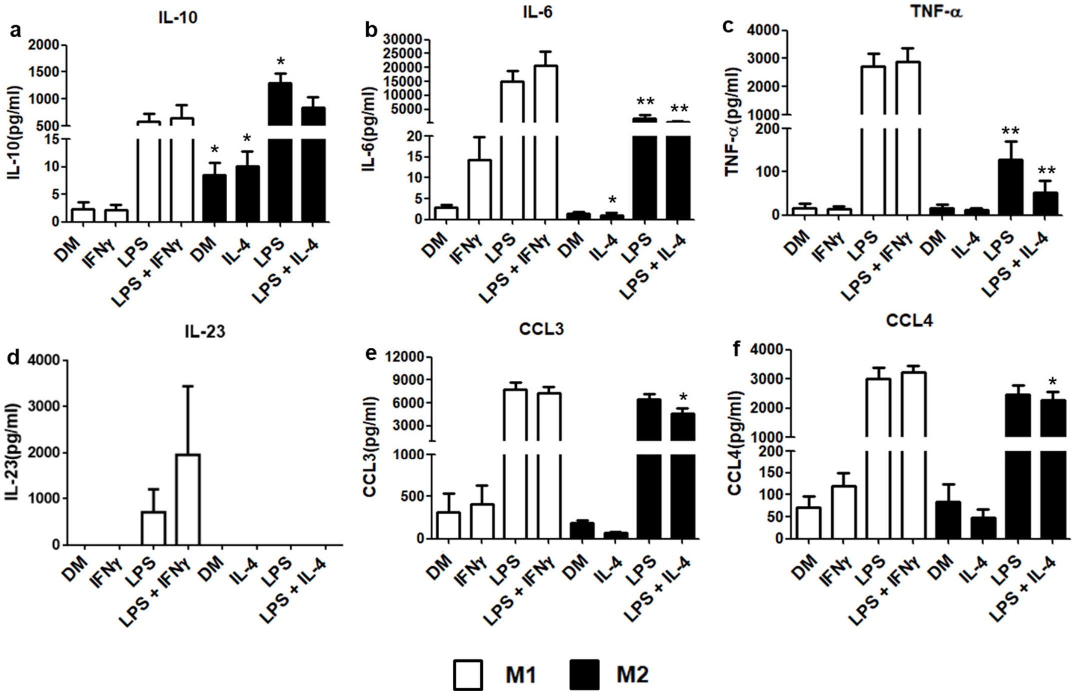

Figure S2. Secretion of cytokines/chemokines by M1 (white bars) and M2 (black bars) macrophages activated with different stimuli. Culture supernatants were harvested and examined for IL-10 (**a**), IL-6 (**b**), TNF-α (**c**), IL-23 (**d**), CCL3 (**e**), and CCL4 (**f**) production by ELISA. Results are expressed as

pg/ml and represent the mean ± SEM of 5–6 experiments. Comparison of M1 with corresponding M2 cells: * $p < 0.05$; ** $p < 0.01$ unpaired T-test. DM: Differentiation media.

Table S1. Migration of M1 and M2 macrophages activated with different stimuli in response to either CCL7 or OPN. Results are expressed as the number of migrating cells and are the mean±SEM of the results from 3–22 experiments. Comparison between M1 and corresponding M2 cells: * $p <$ 0.0.5, ** $p < 0.01$, *** $p < 0.0001$, unpaired T test.

M1 cells			
**Chemoattractant**	**IFNγ**	**LPS**	**LPS + IFNγ**
CCL7	122 ± 17	123 ± 21	146 ± 27
OPN	118 ± 26	130 ± 26	182 ± 57
**M2 cells**			
**Chemoattractant**	**IL-4**	**LPS**	**LPS + IL-4**
CCL7	163 ± 25***	101 ± 26**	168 ± 44
OPN	163 ± 43 (p=0.06)	193 ± 42**	142 ± 48
